# Action Biases Perceptual Decisions Toward Expected Outcomes

**DOI:** 10.1037/xge0000826

**Published:** 2020-12-07

**Authors:** Daniel Yon, Vanessa Zainzinger, Floris P. de Lange, Martin Eimer, Clare Press

**Affiliations:** 1 Department of Psychological Sciences, Birkbeck, University of London, and Department of Psychology, Goldsmiths, University of London; 2 Department of Psychological Sciences, Birkbeck, University of London; 3 Donders Institute for Brain, Cognition, and Behavior, Radboud University; 4 Department of Psychological Sciences, Birkbeck, University of London

**Keywords:** action, perception, prediction, sensorimotor, modeling

## Abstract

We predict how our actions will influence the world around us. Prevailing models in the action control literature propose that we use these predictions to suppress or “cancel” perception of expected action outcomes, to highlight more informative surprising events. However, contrasting normative Bayesian models in sensory cognition suggest that we are more, not less, likely to perceive what we expect—given that what we expect is more likely to occur. Here we adjudicated between these models by investigating how expectations influence perceptual decisions about action outcomes in a signal detection paradigm. Across three experiments, participants performed one of two manual actions that were sometimes accompanied by brief presentation of expected or unexpected visual outcomes. Contrary to dominant cancellation models but consistent with Bayesian accounts, we found that observers were biased to report the presence of expected action outcomes. There were no effects of expectation on sensitivity. Computational modeling revealed that the action-induced bias reflected a sensory bias in how evidence was accumulated rather than a baseline shift in decision circuits. Expectation effects remained in Experiments 2 and 3 when orthogonal cues indicated which finger was more likely to be probed (i.e. task-relevant). These biases toward perceiving expected action outcomes are suggestive of a mechanism that would enable generation of largely veridical representations of our actions and their consequences in an inherently uncertain sensory world.

Effectively acting on the world around us requires predicting the consequences of our actions (James, 1890). We select actions based on their predicted outcomes and use these predictions to generate rapid corrective movements when we experience deviant sensory input (Hommel, Müsseler, Aschersleben, & Prinz, 2001; Wolpert, Ghahramani, & Jordan, 1995). Influential “Cancellation” models in the action control literature propose that we also use these predictions to suppress perception of expected sensory inputs across sensory modalities (Bays & Wolpert, 2007; Blakemore, Wolpert, & Frith, 1998; Fiehler, Brenner, & Spering, 2019; Kilteni & Ehrsson, 2017; Kilteni, Houborg, & Ehrsson, 2019; see also Müsseler & Hommel, 1997; Figure 1a). Such a mechanism would allow us to ignore predictable sensations and therefore remain maximally sensitive to more behaviorally relevant unexpected events. Such cancellation models provide an appealing explanation for why it is difficult to tickle oneself (Weiskrantz, Elliott, & Darlington, 1971). The idea has also drawn wide support from studies showing that sensory events predictably resulting from action are perceived as less intense than similar events presented in the absence of action (Bays, Wolpert, & Flanagan, 2005; Sato, 2008). Indeed, interest in cancellation mechanisms has been galvanized by studies suggesting an intimate link between such effects and the feelings of control that accompany out movements (the “sense of agency”), with dysfunctions of cancellation associated with pathologies of agency in a variety of psychiatric conditions (Frith, Blakemore, & Wolpert, 2000).[Fig-anchor fig1]

However, the core principle guiding Cancellation models—that perception of predicted inputs is suppressed—contrasts with prominent Bayesian models in the wider sensory cognition literature (Figure 1b). These models suggest that we are more, not less, likely to perceive what we expect (de Lange, Heilbron, & Kok, 2018; Press & Yon, 2019). They emphasize how in an inherently ambiguous sensory world, it is adaptive for organisms to combine sampled sensory evidence with prior knowledge about what is likely to occur. Mechanistically this can be achieved by altering the weights on sensory channels, by increasing the “gain” of expected relative to unexpected signals (de Lange et al., 2018; Summerfield & de Lange, 2014). Increasing the gain afforded to expected sensory signals in this fashion—effectively “turning up the volume” of events that conform to our prior predictions—would bias perceptual processing and predispose observers to perceive events that they expect to occur (e.g., Hudson, Nicholson, Ellis, & Bach, 2016; Hudson, Nicholson, Simpson, Ellis, & Bach, 2016; Wyart, Nobre, & Summerfield, 2012). For example, a range of curious illusory phenomena—such as the tendency of observers to perceive concave faces as convex (Gregory, 1997)—could arise via such mechanisms. Importantly, while these kinds of biases may lead to occasional misperceptions, they may nonetheless reflect an adaptive and efficient way of generating veridical percepts, since expected events are, by definition, more likely to occur. While these models have been developed outside of action contexts, a mechanism that biases perception in line with expectations could be just as adaptive during action. For example, if we are trying to flick the light switch in a darkened room, we will generate more veridical estimates of our ongoing actions if we are biased to perceive expected events (e.g., the sight of a moving hand).

Cancellation and Bayesian accounts of how predictions shape perception have been difficult to compare directly because experimental approaches differ between disciplines (Press, Kok, & Yon, 2020b). Support for Bayesian models within the normative sensory cognition literature typically examines an organism’s ability to detect a low intensity stimulus (Stein & Peelen, 2015; Wyart et al., 2012). In contrast, action studies reporting cancellation have typically asked participants to judge the intensity of action outcomes (Bays et al., 2005; Blakemore et al., 1998; Kilteni et al., 2019; Sato, 2008; Weiss, Herwig, & Schütz-Bosbach, 2011; see also Yon & Press, 2017, 2018). To render the paradigms more comparable, the present series of perceptual experiments used a signal detection paradigm within the domain of action (see also Cardoso-Leite, Mamassian, Schütz-Bosbach, & Waszak, 2010; Schwarz, Pfister, Kluge, Weller, & Kunde, 2018). They also presented visual action outcomes, given that both Cancellation and Bayesian theories hypothesize comparable operation of mechanisms across sensory modalities (Brown, Adams, Parees, Edwards, & Friston, 2013; Wolpert, Doya, & Kawato, 2003) and that visual events have been used more commonly in the normative sensory cognition literature (see General Discussion).

Participants produced manual actions (abducting their index or middle finger) and detected visual action outcomes, which could be congruent or incongruent with their own movement. This congruency manipulation exploits the fact that congruent action outcomes will be more expected than incongruent ones, based either on inherited evolutionary expectations or our extensive experience of controlling our actions (Hommel et al., 2001). Under Cancellation models, suppressing activity in units tuned to expected stimuli should make it harder for predictable action outcomes to reach detection threshold (see Figure 1), making observers either less sensitive to these events or biased to report that they did not occur. In contrast, under normative Bayesian models selectively increasing the relative weight on expected sensory channels should either bias observers to report that congruent events occurred or make them more sensitive to congruent outcomes. However, given recent findings in the broader sensory cognition literature, we anticipated that sensitizing sensory channels tuned to expected events would increase hits and also false alarms—in signal detection terms, biasing observers rather than increasing their sensitivity (Wyart et al., 2012). We subsequently used computational modeling to pinpoint which aspect of perceptual decision making is influenced by expectations.

## Experiment 1: How do Actions Influence Detection of Expected Outcomes?

### Method

#### Participants

Twenty-four healthy participants (19 female, 5 male, mean age = 24.9 years, *SD* = 5.38) took part in Experiment 1. All participants in all experiments reported normal or corrected-to-normal vision and no history of psychiatric or neurological illness. A sample size of 24 participants per experiment was selected such that each would have at least 80% power to detect a medium-sized effect of action-outcome congruency on perceptual decisions (Cohen’s *dz* = .6, *N* = 24, α = .05 provides 80.3% power - G*Power 3.1.9.2). All experiments were approved by the local ethics committee at Birkbeck, University of London.

#### Procedure

The experiment took place in a dimly lit testing cubicle. Participants sat ∼55 cm from the monitor (153 × 32 cm, 60 Hz) used for stimulus presentation, with their hands placed above two keypads. The participant’s right hand was rotated 90°, such that their knuckles were aligned with the body midline. Each trial began with the presentation of a greyscale avatar hand (Poser 10, Smith Micro Software). This image remained on screen until participants executed either an index or middle finger tapping action—depressing the relevant key. Movements were freely selected, but the experiment ran until participants executed at least 100 of each type. On 50% of trials, participant’s actions triggered a synchronous movement of the onscreen hand (signal present) that was displayed for 17 ms. On the remaining 50% of trials, the hand remained still (signal absent). On signal present trials, half of observed movements were congruent with the participant’s own action (e.g., execute index tap, observe index tap), and half were incongruent (e.g., execute index tap, observe middle tap). Regardless of signal presence, the index and middle region of the avatar hand was backward-masked by an oval texture comprised of avatar fingers (Cutting, Moore, & Morrison, 1988) for 100 ms. This image was in turn followed by a visual white noise mask presented for 300–600 ms. Participants were subsequently asked about the movement of one of the two fingers (e.g., did the INDEX finger move?). They registered their decision with a button press with their left thumb. On half of the trials, participants were probed about the congruent finger of the avatar hand (i.e. the index finger if they moved their index finger), and on the remaining half, the incongruent finger was probed. Participants in Experiment 1 also made a confidence judgment about their decision (“high confidence” or “low confidence”) to collect pilot data for an additional experiment.

Participants completed at least 200 trials.^1^ Trial types were randomized across the experiment, and breaks were taken every 40 trials. Before the main experiment, participants completed a short practice block that familiarized them with the main task (16 trials). Participants subsequently completed a longer practice block without producing actions where they completed a 1 up–1 down adaptive staircase to adjust the difficulty of the perceptual discrimination such that it was approximately matched for all participants. This staircase targeted the amount of observed finger movement (minimum 1° rotation around the metacarpophalangeal joint, maximum 16°) that was required for detection on ∼50% of trials. The staircase terminated after 12 reversals, and the average of the last six turning points was taken as an estimate of the participant’s threshold. The main experiment began after completing the adaptive staircase, and test stimuli (when present) were shown at this threshold value on all subsequent trials.

### Results and Discussion

All tests in all experiments used an alpha level of .05, and for nonsignificant results we calculated Bayes Factors to quantify evidence for the absence of an effect (i.e. the null hypothesis; Dienes, 2014). Separate signal detection theoretic measures of sensitivity (*d*′) and bias (*c*) were calculated using hit rates and false alarm rates on congruent and incongruent trials. *d*′ reflects the extent to which participants are more likely to report the presence of a stimulus when it is present than when it is absent (*d*′ = z[hit rate] – *z*[false alarm rate]), while *c* reflects the extent to which participants are more likely to respond “present” or “absent” regardless of objective stimulus presence (*c* = −.05[z(hit rate) + z(false alarm rate)]). For some participants in some conditions, response counts were empty (e.g., no misses), which can preclude calculation of *d*′ and *c.* In line with previous recommendations (Hautus, 1995), this issue was overcome by adjusting counts of hits, misses, false alarms, and correct rejections by +.5 in all experiments, and this adjustment was applied to all participants to avoid introducing biases into group-level analyses (Snodgrass & Corwin, 1988).

**Figure 2 fig2:**
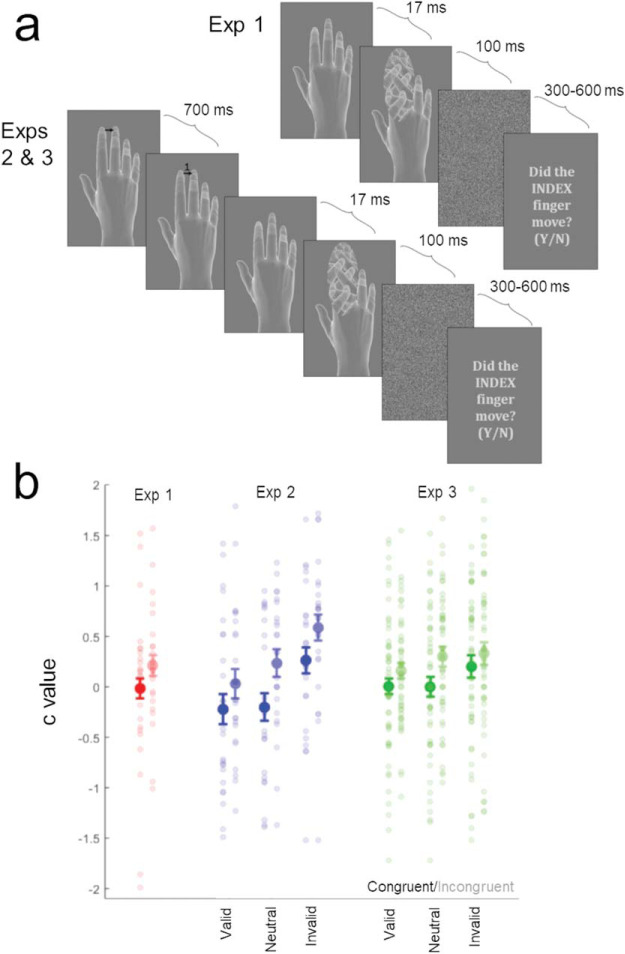
Action execution and detection task, with signal detection *c* results from Experiments 1–3. a. Participants performed actions, which were paired with synchronous congruent or incongruent movements of an avatar hand that they were required to detect. In Experiments 2 and 3, attentional arrow cues also informed participants about which finger of the avatar hand was likely to be probed. b. We calculated the signal detection theoretic measure *c* to index biases induced by action. These values were lower (i.e. responses were more liberal) on congruent (saturated) relative to incongruent (desaturated) trials, irrespective of attentional focus. This effect demonstrates that perceptual decisions were biased toward expected action outcomes. Error bars show 95% within-participant confidence intervals of the mean difference between conditions.

Sensitivity and bias on congruent and incongruent trials were compared using *t* tests. These analyses revealed that participants were more liberal in reporting the presence of congruent action outcomes (lower *c* – *t*_23_ = 2.35, *p* = .028, *dz* = .480; see Figure 2) and were also more sensitive when judgments probed the congruent finger (higher *d*′ – *t*_23_ = 2.29, *p* = .031, *dz* = .467; see Table 1). These findings are predicted by the Bayesian account and inconsistent with the Cancellation account.[Fig-anchor fig2][Table-anchor tbl1]

## Experiment 2: Dissociating Effects of Expectation and Attention on Detection Performance

Experiment 1 found that participants were more sensitive to (higher *d*′) and biased to report the presence of (lower *c*) congruent action outcomes. However, one possibility is that these results reflect effects of “attention” rather than “expectation” per se. That is, in both laboratory tasks and natural settings, top-down expectations (i.e. what is likely to occur) are often confounded with top-down attention (i.e. what is relevant for task performance; Summerfield & Egner, 2016). While in our task movements of congruent fingers are just as probable as incongruent ones—making both types of event equally task-relevant—actors may have learned outside the laboratory to allocate top-down attention to congruent fingers as these are typically more relevant for controlling our actions (note of course that our logic also assumes that congruent movements are more expected due to learning outside of the experimental setting; see Introduction). This interpretation is particularly likely, given that when expectation and attention have been orthogonalized in the sensory cognition literature, the former has been found to generate biasing effects and the latter sensitivity effects (Wyart et al., 2012). Experiment 2 examined this possibility by orthogonally manipulating action-outcome congruency and task relevance with a new sample. This was achieved by introducing two separate cues to the task: a number cue indicating which action participants should perform (thereby manipulating expectations about outcomes) and an orthogonal arrow cue indicating which outcomes will be relevant for perceptual decisions (indicating what participants should attend to).

### Method

#### Participants

A new sample of 24 participants took part in Experiment 2 (17 female, 7 male, mean age = 24.4 years, *SD* = 4.23). Data from one additional participant was lost due to a technical malfunction.

#### Procedure

The procedure of Experiment 2 was identical to Experiment 1 with the following changes. Trials began with the presentation of the neutral observed hand overlaid with an arrow cue. On 50% of trials, the arrow cue was valid, pointing to the finger that was subsequently probed. On 25% of trials the cue was invalid, pointing to the finger that was not subsequently probed. On the remaining 25% of trials, the cue was neutral, pointing to both fingers and indicating that they would be probed with equal probability. After 700 ms, an imperative cue (“1” or “2”) was presented above the arrow, indicating which action participants were required to perform (index or middle tap, respectively). After participants executed the correct action, the same stimulus sequence was triggered as in Experiment 1. Participants made the same detection judgments, but confidence judgments were not collected in this experiment. The experiment comprised 320 trials. Trial types were randomized across the experiment, and breaks were taken every 20 trials.

### Results and Discussion

Signal detection theoretic measures of sensitivity and bias were calculated for each combination of action-outcome congruency (congruent, incongruent) and cue validity (valid, neutral, invalid), and effects were evaluated using ANOVAs with the same factorial structure. Both analyses revealed an effect of cue validity, such that participants were more sensitive to (higher *d*′ − *F*_2,46_ = 4.246, *p* = .020, η_*p*_^2^ = .156; Table 1) and more liberal in reporting (lower *c − F*_2,46_ = 14.57, *p* < .001, η_*p*_^2^ = .388; Figure 2) validly cued events—validating that participants used these cues to guide their attention (Hawkins et al., 1990). *T* tests decomposing these cuing effects found that judgments were more sensitive when cues were valid than invalid (*t*_*23*_ = 2.85, *p* = .009, *dz* = .581), though differences between sensitivity on valid and neutral cuing trials (*t*_*23*_ = 1.98, *p* = .059, *dz* = .404, *BF*_01_ = 1.36) or neutral and invalid trials (*t*_*23*_ = .937, *p* = .359, *dz* = .191, *BF*_01_ = 3.01) were nonsignificant. Comparable tests found observers were more liberal on validly cued trials than invalid ones (*t*_*23*_ = 4.11, *p* < .001, *dz* = .838) and more liberal on neutrally cued than invalidly cued trials (*t*_*23*_ = 4.81, *p* < .001, *dz* = .981), but differences between valid and neutral cuing trials were not significant (*t*_*23*_ = 1.28, *p* = .214, *dz* = .261, *BF*_01_ = 2.21). Moreover, effects of cue validity on judgment sensitivity did not interact with action-outcome congruency (*F*_2,46_ = .184, *p* = .833, η_*p*_^2^ = .008, *BF*_01_ = 7.19).

Importantly, these analyses also found that participants were biased to report the presence of congruent action outcomes (*F*_1,23_ = 8.47, *p* = .008, η_*p*_^2^ = .269)^2^, and the magnitude of this bias did not interact with the focus of attention (*F*_2,46_ = 1,29, *p* = .286, η_*p*_^2^ = .053, *BF*_01_ = 5.95), suggesting that observers are biased to perceive predictable action outcomes irrespective of task relevance. However, there was no significant effect of congruency on sensitivity (*F*_1,23_ = 1.70, *p* = .205, η_*p*_^2^ = .069, *BF*_01_ = 1.52). Therefore, when attention is orthogonally manipulated, participants are still biased (lower *c*) to report the presence of congruent action outcomes. The effect of congruency on sensitivity was not detectable in Experiment 2, consistent with the possibility that this effect in Experiment 1 was determined by attentional processes (Wyart et al., 2012).

## Experiment 3: Controlling Imperative-Outcome Mapping

Experiment 3 investigated whether the congruency biasing effect was driven by expectations engendered by action or a mapping between imperative cues and stimulus types (1 = index/left; 2 = middle or right—a common mapping used in musical training). To rule out possible mapping between cues and outcomes, we compared one group of participants on an identical procedure as Experiment 2 to another group who received arbitrary shapes as imperatives rather than numbers (NB: no cue-outcome mapping can be learnt within the experiment as there is no within-experiment contingency).

### Method

#### Participants

A new sample of 48 participants took part in Experiment 2 (33 female, 15 male, mean age = 24.7 years, *SD* = 4.08), with 24 in each of the two groups. This provides the same power to detect the effect individually within each of the two groups as in Experiments 1 and 2, as well as enabling examination of the interaction according to cue type.

#### Procedure

One group of 24 participants completed a procedure identical to Experiment 2, where numbers (“1” or “2”) cued participants to perform index or middle finger actions. A separate group of 24 participants completed a near identical procedure, except circles and squares indicated to participants that they should execute index and middle finger movements. Half of these participants received a circle-index/square-middle mapping, and half received a circle-middle/square-index mapping.

### Results and Discussion

Measures of sensitivity and bias were calculated separately for each combination of experimental conditions and analyzed with separate ANOVAs. Therefore, the only change with respect to Experiment 2 was the addition of a between-participants factor of cue type.^3^ Greenhouse Geisser corrections were employed where appropriate. Participants were again more liberal in reporting the presence of congruent than incongruent action outcomes (*F*_1,46_ = 14.59, *p* < .001, η_*p*_^2^ = .241; Figure 2) and more liberal in reporting the presence of validly cued events (*F*_2,92_ = 5.062, *p* = .008, η_*p*_^2^ = .009). *T* tests decomposing the attentional cuing effect on *c* values found that observers were more liberal on validly cued trials than invalid ones (*t*_47_ = 2.73, *p* = .009, *dz* = .394) and more liberal on neutrally cued than invalidly cued trials (*t*_47_ = 2.47, *p* = .017, *dz* = .356), but differences between valid and neutral cuing trials were not significant (*t*_47_ = 1.19, *p* = .242, *dz* = .171, *BF*_01_ = 3.30).

The effect of action-outcome congruency did not interact with the focus of attention (*p* = .080, η_*p*_^2^ = .025, *BF*_01_ = 3.81). Crucially, cue type (shapes vs. numbers) also did not interact with the factor action-outcome congruency (*F*_1,46_ = 1.192, *p* = .281, η_*p*_^2^ = .025, *BF*_01_ = 2.61) or generate a three-way interaction with congruency and attentional focus (*F*_2,92_ = .234, *p* = .792, η_*p*_^2^ = .005, *BF*_01_ = 7.69). Indeed, separate analyses of the number (*F*_1,23_ = 7.50, *p* = .012, η_*p*_^2^ = .246) and shape cuing conditions (*F*_1,23_ = 8.02, *p* = .009, η_*p*_^2^ = .259) revealed a significant effect of congruency in both groups—underscoring that participants were more likely to report the presence of congruent action outcomes irrespective of cue type.

Equivalent analyses of *d*′ found that judgment sensitivity again improved with valid cues – (*F*_2,92_ = 9.87, *p* < .001, η_*p*_^2^ = .257; Table 1). *T* tests decomposing this cuing effect found judgments were more sensitive when cues were valid compared to invalid (*t*_47_ = 4.49, *p* < .001, *dz* = .648), valid compared to neutral (*t*_47_ = 2.42, *p* = .019, *dz* = .349), and neutral compared to invalid (*t*_47_ = 2.07, *p* = .044, *dz* = .298). However, *d*′ was unaffected by action-outcome congruency (*F*_1,46_ = .092, *p* = .763, η_*p*_^2^ = .002, *BF*_01_ = 7.63), and effects of relevance did not interact with congruency (*F*_2,92_ = .638, *p* = .531, η_*p*_^2^ = .014, *BF*_01_ = 9.80). Neither the main effect of validity (*F*_2,92_ = .194, *p* = .824, η_*p*_^2^ = .004, *BF*_01_ = 11.7) nor any interaction with congruency (*F*_2,92_ = .542, *p* = .583 η_*p*_^2^ = .012, *BF*_01_ = 6.21) was affected by cue type. Experiment 3 therefore replicated the findings from Experiment 2 while controlling for the particular imperative mapping, suggesting that it was the action-based expectations that generated the biasing effects.

### Combined Analysis

No significant congruency effects on *d*′ were found in Experiments 2 and 3, in contrast to Experiment 1 where observers were more sensitive to the presence of congruent action outcomes. As noted, one plausible explanation for this discrepancy is that effects on sensitivity are driven by attentional mechanisms (see Wyart et al., 2012), and sensitivity effects were correspondingly abolished when attention was orthogonally cued in Experiments 2 and 3. However, Bayes Factors suggested evidence for a null result was convincing in Experiment 3 (*BF*_01_ > 3) but indecisive in Experiment 2 (*BF*_01_ < 3). Combining data from Experiments 2 and 3 for greater precision, we found convincing evidence for the absence of a congruency effect on *d*′ - *t*_71_ = 1.02, *p* = .312, *dz* = .120, *BF*_01_ = 4.69, suggesting that sensitivity effects were absent across the two experiments.

### Computational Modeling: Which Aspect of Perceptual Decision Making Is Biased by Action?

**Figure 3 fig3:**
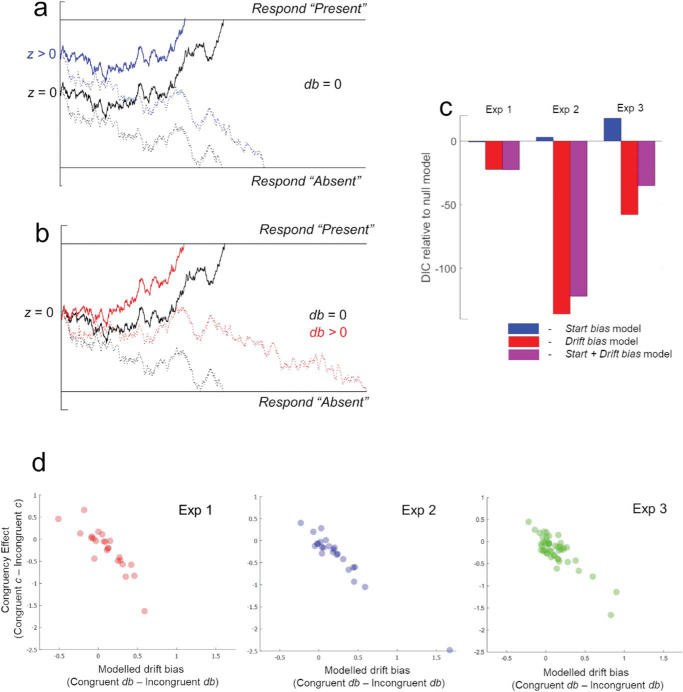
Illustration of how the DDM could explain action-induced biases, and results of computational modeling. a. For an unbiased decision process (black lines) sensory evidence integrates toward the upper response boundary when stimuli are present (solid lines) and toward the lower response boundary when stimuli are absent (dotted lines). Baseline shifts in decision circuits could shift the start point of the accumulation process nearer to the upper boundary for congruent events (influencing the parameter *z;* blue lines – *Start bias* model). b. Alternatively, selectively altering the weights on sensory channels could bias evidence accumulation in line with expectations (influence parameter *db;* red lines – *Drift bias* model). c. Across all experiments, the *Drift bias* model provided a better fit than the *Start bias* model (lower deviance information criteria [DIC] indicates better model fit). d. Moreover, in each experiment, there was a strong correlation between the drift bias values modeled to each participant and the empirical action-induced bias on *c* values.

We used computational modeling of participant choices and reaction time (RT) distributions to pinpoint which aspect of perceptual decision making is biased by action. Drift diffusion models (DDMs) of perceptual decision making have enjoyed growing prominence in the cognitive sciences (Ratcliff, Smith, Brown, & McKoon, 2016; see Figure 3). These models assume that when making perceptual choices (e.g., was a stimulus present or not?), observers have an internal representation of “sensory evidence” that is sampled by decision circuits. Decision circuits continuously sample from the representations of sensory evidence, and when the accumulated decision variable meets a response boundary (e.g., ‘respond present’), the appropriate response is triggered. The representation of sensory evidence is therefore separable from the representation of decisions about that evidence.[Fig-anchor fig3]

There are two ways that the DDM could accommodate the action-induced bias found in Experiments 1–3. First, expectations during action could shift the starting point of the evidence accumulation process toward the “respond present” decision bound (varying parameter *z* of the DDM; see Figure 3a). Such effects are often thought to reflect biases in the decision circuits. For example, predictive cues can induce preparatory motor activity before a stimulus is presented (de Lange, Rahnev, Donner, & Lau, 2013), and these starting point effects could operate even if participants are insensitive to the perceptual information (e.g., they closed their eyes). However, a second alternative is that action directly biases sensory representations, as though agents have selectively increased the gain (or “precision”) afforded to expected sensory signals (Friston, 2018). This kind of bias would manifest as an asymmetric bias in the rate of evidence accumulation (Mulder, Wagenmakers, Ratcliff, Boekel, & Forstmann, 2012; “drift biasing,” affecting parameter *db* of the DDM; see Figure 3b) because, as the sensory evidence is sampled more, it provides progressively more evidence in favor of the expected event.

### Method

To investigate whether either of these possibilities could account for the action-induced bias we observed in our experiments, we fit hierarchical DDMs to participant choice and RT data using the hDDM package implemented in Python (Wiecki, Sofer, & Frank, 2013). In the hierarchical DDM, model parameters for each participant are treated as random effects drawn from group-level distributions, and Bayesian Markov Chain Monte Carlo (MCMC) sampling is used to estimate group and participant level parameters simultaneously.

We specified four different models for data in each experiment: (a) a *null* model where no parameters were permitted to vary between congruent and incongruent trials; (b) a *start bias* model where the start point of evidence accumulation (*z*) could vary on congruent and incongruent trials; (c) a *drift bias* model where a constant bias in evidence accumulation (*db*) could vary between congruent and incongruent trials; (d) a *start + drift bias* model where both parameters could vary. To improve model fits, all models in Experiments 2 and 3 also allowed global drift rate (but not drift bias—i.e. biases to drift asymmetrically toward present or absent decisions) to vary as a function of cue validity to account for the effect of attentional cues on *d*′ (Ratcliff et al., 2016). Varying drift rate (rather than drift bias) captures effects that reflect more reliable evidence accumulation to the appropriate response boundary (i.e. “respond present” when stimuli are present and “respond absent” when absent) and therefore accounts for sensitivity effects seen as a function of cue validity, rather than a biased accumulation toward one response boundary over another. In no model did we allow drift rate to vary between congruent and incongruent trials, as this would amount to assuming that observers are generally more sensitive to all kinds of sensory evidence on congruent trials (e.g., higher *d*′), rather than sensitizing particular sensory channels that encode expected outcomes.

All models were estimated with MCMC sampling with 30,000 samples (“burn-in” = 7500). Model convergence was assessed by inspecting chain posteriors and simulating RT distributions for each participant. Models were compared using deviance information criteria (DIC) as an approximation of Bayesian model evidence. Estimated parameters in each model were compared using the Bayesian significance test implemented in hDDM, which computes the posterior probability that group-level parameters differ across conditions.

### Results and Discussion

Fitting the DDM to the behavioral data found—in all experiments—that the drift biasing model provided a better fit than the start biasing model and that in Experiments 2–3 a model only implementing a drift bias outperformed a model implementing both biases (see Figure 3c). Analyzing modeled parameters revealed higher drift biases on congruent relative to incongruent trials (posterior probabilities that congruent *db* > incongruent *db*: Experiment 1 = 0.819, Experiment 2 = 0.959, Experiment 3 = 0.899—higher values indicate greater differences between conditions; Wiecki et al., 2013). To confirm that these differences in drift bias explained the effect of expectations on perceptual decisions, we calculated and correlated the difference between drift bias parameters (congruent *db* – incongruent *db*) and the magnitude of the behavioral bias (congruent *c* – incongruent *c*) for each participant. This analysis revealed strong relationships within all three samples (Experiment 1: *r*_24_ = −.877, *p* < .001; Experiment 2: *r*_24_ = −.973, *p* < .001; Experiment 3: *r*_48_ = −.855, *p* < .001, see Figure 3d).

This modeling thereby suggests that action-induced biases were best accounted for by a sensory drift biasing mechanism—where observers are biased to accumulate sensory evidence in line with their expectations—rather than a change in later decision circuits.

## General Discussion

Cognitive scientists have proposed models of perceptual prediction that disagree about how our expectations should shape what we perceive (Press et al., 2020b). Cancellation models influential in action control have suggested that agents are less likely to perceive the predictable consequences of their actions (Bays & Wolpert, 2007), in contrast to Bayesian models from the wider sensory cognition literature that suggests observers weight perception toward prior knowledge (see de Lange, Heilbron, & Kok, 2018; Press & Yon, 2019). The present experiments suggest that evidence accumulation is biased in line with expectations during action such that observers are more likely to perceive the outcomes they expect. This pattern concords with Bayesian accounts of expectation developed in the wider sensory cognition literature, which assume observers increase the weight they give to expected inputs when making perceptual judgments. Biasing perceptual decisions in this fashion is an effective way to rapidly generate more veridical percepts from sensory signals corrupted by irreducible internal and external noise. While this process increases the likelihood that expected signals are detected (i.e. more hits), it also makes observers prone to hallucinate events when confronted with signal-like noise (i.e. more false alarms; Wyart et al., 2012) and therefore is thought to generate biasing rather than sensitivity effects—as observed here. Indeed, we found these action-induced biases in perception were dissociable from top-down attention, with orthogonal task-relevance cues altering judgment sensitivity. This finding is in line with previous studies of expectation and attention outside of action contexts (Wyart et al., 2012) and perhaps therefore indicative of domain-general mechanisms.

These patterns are consistent with findings of how previous decisions about sensations influence subsequent decisions (Talluri, Urai, Tsetsos, Usher, & Donner, 2018) and with a recent fMRI study that found that expected action outcomes are more readily decoded from early and late visual brain areas (Yon, Gilbert, de Lange, & Press, 2018). These neural effects are predicted by Bayesian models, but it has alternatively been suggested that effects of expectation in sensory brain areas could reflect feedback from decision-related areas in higher-level cortex that have no causal effect on perception (Bang & Rahnev, 2017; Choe, Blake, & Lee, 2014). The current findings importantly indicate effects of action expectation on perception and a corresponding drift biasing mechanism that is consistent with an early sensory biasing account.

The predictive relationship exploited in these experiments—for example, that observed index finger movements are an expected consequence of moving one’s index finger—is strong and stable. It reflects an expectation that is likely acquired through our extensive experience of controlling our actions (Hommel et al., 2001). However, we would predict that in principle the same underlying mechanisms operate when we acquire new predictions. In line with this assumption, our effects are akin to those found—outside of action settings—when participants learn within an experiment that color cues predict the orientation of gratings (Wyart et al., 2012). The hypothesis that similar mechanisms operate when we acquire new predictions may appear inconsistent with previous conflicting findings when participants are presented with novel action-outcome mappings within an experiment. In an elegant training study, Cardoso-Leite et al. (2010) found evidence that participants are less sensitive to grating orientations “predicted” by actions that had been paired with the gratings during training, with no influence of actions on bias measures. However, sensitivity was especially high in this study, such that a bias toward perceiving the expected, accompanied by ceiling effects on the hit rate, would appear as a sensitivity reduction. Perhaps more importantly, this single study used a small sample, and the findings may not replicate (Schwarz et al., 2018). It may therefore be suggested that there was insufficient opportunity in this paradigm to acquire the action-outcome mappings reliably. However, in principle, given sufficient opportunity for prediction acquisition, we would hypothesize prediction mechanisms to operate in a qualitatively similar fashion when predictions are both “old” and “new” (Dogge, Custers, Gayet, Hoijtink, & Aarts, 2019).

This pattern of results is difficult to reconcile with cancellation models and their central claim that observers are less likely to perceive the multisensory predictable consequences of their movements (Bays & Wolpert, 2007; Blakemore et al., 1998). For example, key support for cancellation models has come from studies that show predictable signals generated by action are perceived to be less intense than similar (i.e. unpredicted) events presented in the absence of action (Bays et al., 2005). These studies are in fact often difficult to interpret because there are several differences between the predicted and unpredicted conditions. Most notably, many of the studies compare perception of “predicted” self-generated events with perception of “unpredicted” sensory events generated by external sources while participants themselves remain passive—or where the sensory and motor events overlap less due to temporal misalignment (Blakemore, Frith, & Wolpert, 1999). This comparison perhaps confounds the operation of expectation mechanisms with that of other processes (see Press, Kok, & Yon, 2020a). For example, if conceptualizing action as an additional task, classic working memory models would hypothesize reduced sensory processing when events are presented in combination with action (Baddeley, 1996). It is therefore difficult to isolate effects of prediction mechanisms from those introduced by the dual- versus single-task design. The measures employed typically also cannot isolate perceptual effects from decisional biases (see Firestone & Scholl, 2016) or expectation from attention-based processes (see Summerfield & Egner, 2016).

One explanation for the difference between the present and previous studies could be that we have examined visual action effects, whereas much evidence to support cancellation comes from tactile paradigms (e.g., Juravle, McGlone, & Spence, 2013; Juravle, Binsted, & Spence, 2017; Kilteni & Ehrsson, 2017). The models of which we are aware hypothesize similar operation of predictive Bayesian or Cancellation mechanisms across domains (Brown et al., 2013; Wolpert et al., 2003), and there may be no reason to hypothesize distinct adaptive arguments for the sense of touch. However, touch may be influenced differently because of operation of additional generalized “suppression” mechanisms. Such suppression mechanisms are thought to attenuate tactile sensations during action regardless of whether they are predicted effects of action or not and are thought to be mediated by spinal mechanisms (Seki & Fetz, 2012), and comparable mechanisms may similarly attenuate sensory processing in a nonpredictive fashion across modalities in humans and other animals (Crapse & Sommer, 2008). Importantly, recent experiments in touch suggest that when confounds related to sensory suppression are removed, action predictions may influence perception in a qualitatively similar fashion irrespective of sensory modality (Thomas, Yon, de Lange, & Press, 2020).

Nevertheless, there remain studies that are less prone to these alternative explanations and report “cancelled” percepts for expected, relative to unexpected, action outcomes (e.g., Roussel, Hughes, & Waszak, 2013, 2014). It is therefore important that future research unpacks the features that have driven previous reports of “cancellation”—including the possibility that additional mechanisms are recruited as sensory processing unfolds (see Press et al., 2020b). Specifically, some of us have recently proposed that the influence of expectation on perception may not be as simple as suggested in either Bayesian or Cancellation theories. Under this proposal, observers are initially biased toward perceiving expected sensory events, but any especially surprising inputs—likely relevant for model updating—are reactively upweighted (Press et al., 2020b). Such a reactive upweighting process only for a subset of “unexpected” events is consistent with evidence from the learning and inference literature and is in line with the proposed adaptive function of a cancellation mechanism—that is, highlighting events that are informative to the organism. This account may explain some discrepancies in the literature—for example, finding perceptual upweighting of rapid, punctate consequences of movement, in contrast with evidence for cancellation-like phenomena when action outcomes unfold dynamically (e.g., Lally, Frendo, & Diedrichsen, 2011). Future studies should therefore establish whether expectation effects are modulated by the time at which perception is probed and the extent of the surprise elicited by an “unexpected” stimulus—which is low with the barely detectable sensations used in the present signal detection task (Press et al., 2020b).

The concept of cancellation has had a wide-ranging influence on research investigating the sense of agency and its aberration in psychiatric disease. For example, experiences of passivity in schizophrenia—where patients feel moved by an external force—have often been attributed to a failure of predictive cancellation, which causes self-produced action outcomes to appear unusually intense (Frith et al., 2000). However, our results suggest that sensorimotor predictions can increase the weight we give to expected sensory signals, which may suggest that these mechanisms contribute to the experience of agency in a different way. Specifically, biases toward perceiving expected outcomes may help observers to overcome sensory noise, giving us higher fidelity representations of our ongoing movements and their consequences. An inability to incorporate this kind of top-down knowledge into perceptual estimates could leave agents with higher levels of uncertainty about their actions, leaving them vulnerable to developing the unusual beliefs that characterize psychosis (Fletcher & Frith, 2009).

Important contributions to work on sensory prediction during action have also come from models of predictive processing and active inference developed in computational neuroscience (Friston, 2005). These models suggest that all aspects of perception, action, and cognition arise as agents minimize the mismatch between their models of the extracranial world and incoming sensory evidence. Notably, this can be achieved either by using the evidence to update the models (i.e. perception) or by using action to change the evidence (i.e. to alter the world so it conforms to the model). In principle, the architecture of these models can account for the tendency of agents to up- or down-weight perception of predictable action outcomes through changes in the gain or “precision” afforded to sensory evidence (Friston, 2008; Yon, de Lange, & Press, 2019). For example, researchers using this framework have suggested that sensory precision on certain channels is attenuated during action—explaining “cancellation” phenomena (Brown et al., 2013; Van Doorn, Paton, Howell, & Hohwy, 2015)—while also suggesting that observers can increase the precision afforded to expected sensory signals—leading to “representational sharpening” and biases toward perceiving what we expect (Friston, 2018). However, while models of precision-weighting during action have important advantages over forward-model based accounts (e.g., where it has been difficult to specify computationally how predictions could “cancel” sensory signals—see Brown et al., 2013), it remains the case that the precision on a sensory channel, and perception of a single event, cannot be up- and down-weighted simultaneously. This makes it difficult to use existing models of active inference to make precise predictions about those conditions under which perception of expected action outcomes should be enhanced and those where it should be attenuated. Our present findings may provide some constraints on such models that aid the generation of more precise behavioral predictions in the future, as these results suggest that the precision afforded to visual channels encoding expected outcomes is augmented rather than attenuated—certainly when sensory events coincide temporally with action initiation. We hope that behavioral experiments like ours will play an important role in informing and constraining such models—elaborating a comprehensive account of how and why sensory gain is augmented and attenuated as we act upon the world around us.

In conclusion, these results have shown that observers are biased toward perceiving the expected outcomes of their movements. These findings are difficult to reconcile with dominant cancellation accounts in action control, but concord well with normative models of Bayesian perceptual inference. Namely, increasing the weight we give to expected sensory signals may explain how we develop a largely veridical representation of our actions and their consequences in an inherently uncertain sensory world.

## Context

Previous work in the lab has investigated whether specific types of sensorimotor representations—that is, visual-motor mirror representations of action—are the product of domain-general statistical learning processes. After finding broad support for this idea (e.g., Cook, Bird, Catmur, Press, & Heyes, 2014; Press et al., 2012), we began to investigate whether domain-general models can explain the functional influence of sensorimotor predictions on perception as well as the origin of such representations. In a recent neuroimaging study (Yon et al., 2018) we found representations in visual brain areas were reshaped toward expected action outcomes, which is more in line with domain-general ideas in the broader sensory cognition literature than the cancellation models in action. However, while these neuroimaging data are consistent with these models, it has been suggested that expectation-related activity in sensory brain areas plays no causal role in determining what we perceive (Bang & Rahnev, 2017; Choe et al., 2014). Here we directly investigated how expectations influence the perception of action outcomes—finding evidence that we are biased toward perceiving expected action outcomes.

## Figures and Tables

**Table 1 tbl1:** Mean (SD) Sensitivity (d′) Values Across All Conditions in Experiments 1–3

Condition	Congruent			Incongruent		
Experiment 1	1.42 (.567)			1.28 (.588)		
	Valid	Neutral	Invalid	Valid	Neutral	Invalid
Experiment 2	0.73 (.532)	0.68 (.539)	0.63 (.455)	0.69 (.536)	0.59 (.537)	0.57 (.521)
Experiment 3	1.14 (.743)	1.05 (.684)	0.90 (.590)	1.14 (.680)	0.993 (.591)	0.923 (.555)

**Figure 1 fig1:**
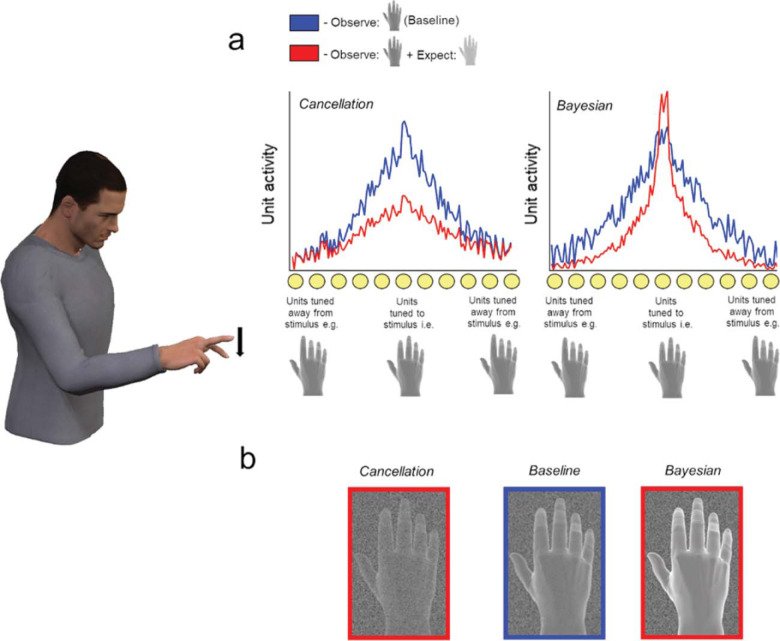
A schematic illustration of how predictive signals influence activation of sensory units under cancellation and Bayesian models, alongside their putative influences on perception. Cancellation models developed in the action literature (a, left) hypothesize that when we move (e.g., depress our index finger), we generate a predictive signal that suppresses activity in sensory units tuned to expected perceptual outcomes (e.g., visual units tuned to the sight of a hand with a depressed index finger). Weakening activity in these units reduces the signal-to-noise ratio of the sensory population—leading to less intense percepts and biasing observers away from perceiving these outcomes (b, left). In contrast, Bayesian models developed in the wider sensory cognition literature (a, right) suggest that predictive signals alter the weights on sensory channels such that the volume is “turned up” (e.g., the gain is increased) on expected relative to unexpected signals. Such weighting leads to a higher signal-to-noise ratio when expectations are valid, leading to more intense percepts and biases toward perceiving expected outcomes (b, right).

## Footnotes

1 Participants in Experiment 1 were successful in following the instruction to execute roughly equal numbers of each action and therefore completed on average 218 trials (*SEM* = 2.5), comprising on average 49.8 % congruent trials (*SEM* = .0014).

2 In Experiment 2, one participant was an outlier, showing an especially large action-induced bias (*z* score = 3.76). Re-running the same analyses without data from this participant revealed the same effect of action congruency on c values (*p* = .003), the same correlation between congruency effects and modeled drift biases (*r* = −.924, *p* < .001) and did not change any other statistical patterns observed.

3 In Experiment 3, one participant was an outlier, showing an especially large action-induced bias (*z* score = 4.12). Rerunning the same analyses without data from this participant revealed the same effect of action congruency on c values (*p* < .001), the same correlation between congruency effects and modeled drift biases (*r* = −.807, *p* < .001) and did not change any other statistical patterns observed.
